# Realizing the World Health Organization’s End TB Strategy (2016–2035): How Can Social Approaches to Tuberculosis Elimination Contribute to Progress in Asia and the Pacific?

**DOI:** 10.3390/tropicalmed4010028

**Published:** 2019-02-05

**Authors:** Christopher A. John

**Affiliations:** 1 Melbourne Medical School, The University of Melbourne, Victoria 3010, Australia; cajohn@student.unimelb.edu.au; Tel.: +61-423-936-931; 2 Melbourne School of Population and Global Health, The University of Melbourne, Victoria 3010, Australia

**Keywords:** tuberculosis, health promotion, social medicine, Asia–Pacific, elimination, End TB Strategy

## Abstract

This review article discusses how social approaches to tuberculosis elimination might contribute to realizing the targets stipulated in the World Health Organization’s (WHO) End TB Strategy (2016–2035), with an emphasis on opportunities for progress in Asia and the Pacific. Many factors known to advance tuberculosis transmission and progression are pervasive in Asia and the Pacific, such as worsening drug resistance, unregulated private sector development, and high population density. This review article argues that *historically successful* social solutions must be revisited and improved upon if current worldwide tuberculosis rates are to be sustainably reduced in the long term. For the ambitious targets laid down in the WHO’s End TB Strategy to be met, biomedical innovations such as point-of-care diagnostics and new treatments for multidrug-resistant tuberculosis (MDR-TB) must be implemented alongside economic, social, and environmental interventions. Implementing social, environmental, and economic interventions alongside biomedical innovations and universal healthcare coverage will, however, only be possible if the health and other government sectors, civil society, and at-risk populations unite to work collaboratively in coming years.

## 1. Introduction

This review article discusses how social approaches to tuberculosis elimination might contribute to realizing the targets laid down in the World Health Organization’s (WHO) End TB Strategy (2016–2035), focusing on opportunities for progress in the Asia–Pacific region.

Of the estimated 1.7 billion people asymptomatically infected with *Mycobacterium tuberculosis* worldwide (said to have latent tuberculosis infection), only 5–15% will progress to active (symptomatic) tuberculosis disease during their lifetime. The likelihood of progression to active disease is higher among those affected by risk factors such as human immunodeficiency virus (HIV) infection, diabetes mellitus, cigarette smoking, excessive alcohol use, and malnutrition [1,2].

Tuberculosis is the ninth leading cause of mortality globally and causes more deaths than any other infectious disease, surpassing HIV/acquired immune deficiency syndrome (AIDS) [2,3]. Tuberculosis caused an estimated 1.3 million deaths among HIV-negative people and a further 374,000 deaths among persons infected with HIV in 2016 [2].

### 1.1. The World Health Organization’s End TB Strategy (2016–2035)

The World Health Organization’s End TB Strategy (2016–2035) aims to end the global tuberculosis epidemic by 2035 [4]. The strategy includes targets to reduce absolute mortality by 95% and incidence by 90% between 2015 and 2035, and to make sure that tuberculosis-affected families no longer have to bear catastrophic tuberculosis-related costs by 2030 [4,5]. The strategy was adopted with full support by the World Health Assembly (WHA) in Geneva in May 2014 and urges governments to offer financing and high-level commitment to facilitate the strategy’s implementation [5,6]. The strategy also emphasizes that intersectoral partnerships are crucial to achieving targets, especially with stakeholders in fields such as immigration, labor, justice, and social protection [5]. The strategy stipulates interim milestones, which were set for 2020, 2025, and 2030 [5].

While the strategy’s long-term vision is to eliminate tuberculosis globally, defined as less than one new tuberculosis case per million people per year, executing the time-bound pledge to “end the global tuberculosis epidemic” would mean reducing the global incidence rate from greater than 1000 new cases per million people in 2015 to less than 100 new cases per million people by 2035 [5].

### 1.2. Social Approaches to Tuberculosis Elimination

The World Health Organization (WHO) acknowledges that countries must pursue social protection and universal health coverage to strengthen their social and health sectors if the strategy’s ambitious targets are to be met [5,6]. The Sustainable Development Goals (SDGs) set by the United Nations (UN) also pledge to end the tuberculosis epidemic by combining biomedical, public health, and socioeconomic interventions with innovation and research [5].

The strategy’s proposed interventions were grouped under three strategic pillars [4,5]. The second pillar focuses on “bold policies and supportive systems” and explicitly encourages governments and other stakeholders to take practical steps to attend to the needs of vulnerable populations and “ensure that tuberculosis is addressed in social protection, poverty alleviation, and related social policy agendas and programs” [4,6]. The WHO was charged with responsibility for monitoring progress toward the strategy’s milestones and 2035 targets [5].

For the ambitious targets laid down in the WHO’s End TB Strategy to be met, biomedical innovations such as point-of-care diagnostics, novel vaccines, new treatments for multidrug-resistant tuberculosis (MDR-TB), and biomarkers to detect latent tuberculosis infection (LTBI) must be implemented alongside economic, social, and environmental interventions [4,7,8,9].

### 1.3. Tuberculosis in Asia and the Pacific

Of the approximately 10.4 million people who fell ill with tuberculosis in 2016, 56% were in five countries: India, Indonesia, China, the Philippines, and Pakistan, in descending order of the number of incident cases in each country [2]. Most incident tuberculosis cases in 2016 were in the WHO southeast Asia region (45%); 17% of new cases in 2016 occurred in the WHO western Pacific region [2]. In 2016, 33% of global tuberculosis deaths in HIV-negative people were in India, which also accounted for 26% of overall global tuberculosis deaths among both HIV-negative and HIV-positive people [2].

Drug-resistant tuberculosis continues to threaten international health security and is especially problematic in Asia and the Pacific [2,10,11,12,13]. In 2016, approximately 47% of new tuberculosis cases with resistance to at least rifampicin, the most effective first-line anti-tuberculosis drug, were in India, China, and the Russian Federation [2].

Discrepancies between tuberculosis incidence and reported cases in 2016 were especially marked in India, Indonesia, the Philippines, Pakistan, Bangladesh, and China [2]. In a similar fashion, discrepancies between tuberculosis incidence and treatment enrolment in 2016 were pronounced in India and China, which together accounted for 39% of the global gap [2]. 

These figures underscore south Asia’s staggering tuberculosis caseload and mortality [10]. Many factors known to promote tuberculosis transmission and progression are rampant in south Asia, including high population density, worsening drug resistance, unregulated private-sector development, rising diabetes mellitus incidence, and high levels of both household and outdoor air pollution [10].

## 2. Discussion

### 2.1. Social Approaches to Improving Health

This section seeks to introduce Frieden’s five-tier health impact pyramid and to use it to discuss how social approaches to tuberculosis elimination compare with other types of public health interventions [14]. This health impact pyramid is described in more detail by Frieden elsewhere, but its key attributes are summarized below [14]. Frieden’s five-tier health impact pyramid attempts to describe the varying impact that different types of public health interventions can have [14]. This model suggests that the most impactful interventions (at the pyramid’s base) deal with health’s economic and social determinants and are so effective because they affect society as a whole and depend less on individuals making healthy choices [14]. While it might seem common sense that “public health involves far more than healthcare”, frameworks that attempt to describe health system structures often underestimate the extent to which society’s operation, composition, and organization affect health [14,15,16].

While proven clinical interventions might lessen morbidity and mortality, unpredictable and inconsistent adherence, imperfect effectiveness, and access constraints can curb their overall impact [14,16]. For example, medication non-adherence is known to be more widespread among patients with chronic and asymptomatic conditions, such as LTBI [14]. Access to clinical care can be limited even in those health systems with universal healthcare coverage and especially in systems without it [14,16].

In a similar way, the need to bring about behavior change through health education reflects society’s failure to establish healthy contexts and is by and large a relatively ineffective strategy, despite this being regarded by some as “the essence of public health action” [14]. Even so, some health educational interventions can have significant impact when delivered consistently on many occasions and might be among only a few public health strategies available to address a given problem [14].

When explicating the health impact pyramid, perhaps Frieden’s most important remark is that, although social and contextual interventions are in general the most impactful, others should certainly not be set aside [14]. Indeed, different interventions might be the most feasible and/or effective for different public health challenges in different contexts [14]. As a result, large-scale, global public health programs should seek to embrace many different types of interventions to maximize “synergy and the likelihood of long-term success” [14].

Although the prevailing worldwide tuberculosis epidemic continues to threaten global health security, past tuberculosis control initiatives typify evidence-based public health practice in many ways [7]. Tuberculosis control initiatives that revolve around maximizing patients’ adherence to treatment regimens were historically effective in high-burden and developing settings [4]. Nevertheless, social, epidemiologic, and environmental contexts also significantly influence tuberculosis rates globally, suggesting that biomedical interventions alone cannot be expected to put an end to tuberculosis [7]. Frieden points out that effective tuberculosis control initiatives must combine practical, evidence-based technical interventions with political commitment [7]. Indeed, “context-changing” intersectoral interventions that oppose the epidemic’s social and environmental drivers are unlikely to be implemented without sustained political support [7,14,17,18].

Frieden’s assertion that “context-changing interventions are the most effective public health interventions to improve population health” holds at least as true for tuberculosis as it does for other public health problems [7]. Changing the social environment to make a person’s “default decision healthier” means, among other initiatives, taking steps to tackle poverty’s economic, social, housing and educational sequels [7,15]. However, making this ostensible panacea a reality is also among public health’s greatest challenges [7,14]. As context-changing interventions by definition disrupt society’s economic and social structures and, thus, both call for and give rise to “fundamental societal transformation”, they are almost always controversial [7,14].

### 2.2. Historically Successful Social Approaches to Tuberculosis Elimination

Before streptomycin was discovered in 1944 and made widely available by the late 1940s, and prior to the advent of pyrazinamide and isoniazid in the early 1950s, *social* interventions that improved nutrition, housing conditions, and living standards were instrumental in reducing the global burden of tuberculosis disease [8,19]. These social solutions were usually implemented in sanatoria that isolated patients from the population at large and provided them with proper nutrition, sunlight, space, rest, and clean air [8]. This management approach was, however, both inaccessible to disadvantaged populations with a high tuberculosis disease burden and costly [8].

These downsides prompted countries such as Chile to implement wide-ranging social reforms designed to lessen the country’s national tuberculosis burden and improve the well-being and health of its workforce in the 1930s [8]. Chile had the world’s highest tuberculosis mortality rate in the 1930s and, thus, developed a 14-point social reform plan that specifically addressed the nation’s social inequalities and sought to raise the living standards of the working class [8]. Measures in this plan included increasing wages, improving working conditions in factories, building affordable housing for workers, legislating to protect workers’ health, mandating unemployment insurance, running anti-alcohol campaigns, and promoting sports [8]. The tuberculosis disease burden in all social classes declined as working conditions in factories improved and public sanitation interventions were introduced [8]. Rising living standards and wealth meant that people could afford to eat healthily and live in more spacious accommodation [8].

While these interventions successfully reduced tuberculosis mortality in Chile throughout the 1940s, biomedical strategies took over once chemotherapy became available in the 1950s [8]. Indeed, biomedical interventions have largely underpinned tuberculosis management and control strategies around the world since then [8]. Although the advent of anti-tuberculosis chemotherapy in the early 1950s brought about rapid declines in tuberculosis rates, progress since the 1960s was inconsistent and sluggish [8]. In fact, reductions at least partly attributable to improved living standards occurred long before chemotherapy was introduced in many settings [8,20].

These historically successful social approaches must be revisited if current global tuberculosis trends are to be sustainably reversed in the long term [8]. Implementing social, environmental, and economic interventions alongside biomedical innovations and universal healthcare coverage will only be possible if the health and other government sectors, civil society, and disadvantaged populations work together [8]. As health’s determinants are multi-factorial, comprehensive health-promoting initiatives must correspondingly involve stakeholders from many different sectors [16,21].

### 2.3. Promoting Health to End the Global Tuberculosis Epidemic

Tuberculosis both causes and results from poverty, giving rise to a “a vicious tuberculosis–poverty cycle” that biomedical strategies alone cannot break [8,22]. Tuberculosis drives poverty by leading to loss of income and out-of-pocket healthcare costs [8]. Risk factors associated with poverty meanwhile make progression to active tuberculosis more likely, impose geographical and financial constraints that reduce access to health systems, and might evoke healthcare provider prejudices [8]. Difficulties accessing healthcare delay diagnosis and treatment, which both prolongs a patient’s infectious period and increases the likelihood of death [8].

Risk factors that make tuberculosis transmission and progression more likely include human immunodeficiency virus (HIV) infection, diabetes mellitus, cigarette smoking, excessive alcohol use, malnutrition, overcrowding, and indoor air pollution [2,8,17]. Perhaps excepting HIV infection, these risk factors are yet to be comprehensively addressed by public health programs in low- and middle-income countries [8].

Technical excellence and political leadership are, thus, both key to ending the global tuberculosis epidemic by 2035 for several reasons [7,17,18]. Firstly, as tuberculosis tends to disproportionately affect marginalized and/or socioeconomically disadvantaged population subgroups that do not usually wield political influence, decision-makers often have little incentive to intervene [7]. Secondly, patients and political leaders alike tend to set aside their tuberculosis concerns once the acute symptoms of active disease subside [7]. Finally, these realities mean that epidemiologic analysis is usually unsupported by political and/or media pressure and, thus, often stands alone as a call to action for decision-makers [7]. Thus, as tuberculosis control programs, as well as being controversial and/or costly, often only benefit those without political influence or those who “are unaware (and, therefore, ungrateful) of the risks they are being protected against”, strong political will is necessary to sustain them for the many years needed to maximize impact [7,17,18].

The importance of consumer and community participation and community capacity-building is being increasingly recognized by health promoters in developed and developing countries alike [23]. The capacity-building philosophy holds that “people and communities can manage their own concerns” when given proper support [23]. Arole et al. assert that the community’s role in health promotion is essential [23]. Denholm recently drew attention to the importance of establishing strong links between communities affected by tuberculosis and researchers, and suggests that, without these, research questions might not address the community’s prime concerns, and research outcomes may be improperly implemented and/or poorly communicated [24].

Digital health interventions also have the potential to transform efforts to end tuberculosis [25]. As patients and caregivers around the world increasingly rely on digital technologies such as the internet, personal computers, and mobile devices to carry out day-to-day tasks, these same technologies could well become key to realizing the WHO’s End TB Strategy [25]. For example, digital technologies might be used to help train a community’s health workforce by enhancing information delivery, to prompt individuals within communities to adopt healthy behaviors, to improve adherence to anti-tuberculosis drug regimens, and/or to enable clinicians to more easily access electronic health records and clinical guidelines [25].

Patients and caregivers have in fact already benefited from various digital technologies designed in recent decades to tackle tuberculosis, such as electronic solutions that have enabled tuberculosis surveillance data to be more easily recorded [25]. These innovations have been successfully scaled up in high-burden, large countries such as China and India in the past, but evidence of impact has usually been lacking [25]. Importantly, implementing these technologies widely *without* also striving to improve internet access in remote, rural, and low-resource settings risks *exacerbating* existing social inequities [25].

### 2.4. Implementing Social Solutions in Asia and the Pacific

Asia and the Pacific have the potential to spearhead global tuberculosis elimination efforts by “demonstrating feasibility” in both remote and densely populated settings alike [10]. Indeed, the End TB Strategy’s time-bound pledge to “end the global tuberculosis epidemic” by 2035 will almost certainly go unfulfilled if significant inroads into Asia’s hefty tuberculosis caseload are not made soon [5,10].

Straightforward infection control measures to protect susceptible patients and healthcare workers in healthcare settings, such as administering symptom-based screening questionnaires to hospital visitors and opening windows to improve ventilation, are not routine in south Asia and must thus be made more widespread throughout the region if tuberculosis transmission is to be curbed [10].

As south Asia is prone to political disruption and natural disasters, contingencies to limit disease spread in these circumstances must also be at the ready [10]. For instance, millions of displaced Nepalese people living in unsanitary, overcrowded shelters following the April 2015 Gorkha earthquake were potentially exposed to tuberculosis and multidrug-resistant tuberculosis organisms [10]. This eventuated despite earmarked funding being available, because logistic constraints meant that basic public health measures could not be implemented [10]. Hundreds of tuberculosis patients also had their treatment courses interrupted on account of drug losses and destroyed tuberculosis clinics [10].

Given its massive patient population and abundance of innovative clinicians, scientists, and public health professionals, Asia and the Pacific are well placed to make significant contributions to progress toward targets set in the WHO’s End TB Strategy [10]. By undertaking operational research to better understand and optimize both clinical and social interventions in remote, rural, and urban environments, and by taking steps to improve logistical and policy frameworks that address the upstream and midstream determinants of tuberculosis, Asian and Pacific countries have the opportunity to lead and champion worldwide efforts to end tuberculosis [10,26].

## 3. Conclusions

This review article attempted to outline how social approaches to tuberculosis elimination might contribute to realizing the targets stipulated in the World Health Organization’s End TB Strategy (2016–2035), with an emphasis on how opportunities for progress in the Asia–Pacific region might be capitalized on.

Historically successful social solutions must be revisited and improved upon if current worldwide tuberculosis rates are to be sustainably reduced in the long term [8].

Implementing social, environmental, and economic interventions alongside biomedical innovations and universal healthcare coverage will, however, only be possible if the health and other government sectors, civil society, and disadvantaged populations manage to successfully work together, united by an unwavering resolve to make tuberculosis history [8,21].
